# Weighted Mutual Information for Aggregated Kernel Clustering

**DOI:** 10.3390/e22030351

**Published:** 2020-03-18

**Authors:** Nezamoddin N. Kachouie, Meshal Shutaywi

**Affiliations:** 1 Department of Mathematical Sciences, Florida Institute of Technology, Melbourne, FL 32901, USA; 2 Department of Mathematics, King Abdulaziz University, Rabigh 21911, Saudi Arabia; mshutaywi@kau.edu.sa

**Keywords:** weighted mutual information, aggregated clustering, kernel k-means, conditional entropy

## Abstract

**Background:** A common task in machine learning is clustering data into different groups based on similarities. Clustering methods can be divided in two groups: linear and nonlinear. A commonly used linear clustering method is K-means. Its extension, kernel K-means, is a non-linear technique that utilizes a kernel function to project the data to a higher dimensional space. The projected data will then be clustered in different groups. Different kernels do not perform similarly when they are applied to different datasets. **Methods:** A kernel function might be relevant for one application but perform poorly to project data for another application. In turn choosing the right kernel for an arbitrary dataset is a challenging task. To address this challenge, a potential approach is aggregating the clustering results to obtain an impartial clustering result regardless of the selected kernel function. To this end, the main challenge is how to aggregate the clustering results. A potential solution is to combine the clustering results using a weight function. In this work, we introduce Weighted Mutual Information (WMI) for calculating the weights for different clustering methods based on their performance to combine the results. The performance of each method is evaluated using a training set with known labels. **Results:** We applied the proposed Weighted Mutual Information to four data sets that cannot be linearly separated. We also tested the method in different noise conditions. **Conclusions:** Our results show that the proposed Weighted Mutual Information method is impartial, does not rely on a single kernel, and performs better than each individual kernel specially in high noise.

## 1. Introduction

Large amounts of data are collected on a daily basis through social media, medical imaging equipments, satellite imagery, surveillance cameras, and many more. Developing advanced methods for summarizing, grouping, and mining these large dataset is in high demand [1]. Cluster analysis is a common unsupervised learning method used to discover underlying patterns for dividing data into different groups. Cluster analysis is performed to discover distinct individuals with similar features within a large population and group them in the same cluster [2]. Clustering has been increasingly used to address multidisciplinary problems as an important step in machine learning [3] and data mining [4]. Cluster analysis plays an important role in many areas such as marketing, medical diagnosis, information retrieval, psychology and social sciences, pattern classification, and many more [5]. Some examples are:

- Marketing: Clustering is used for market segmentation to identify customers with similar profiles for advertising purposes [1].
- Web Browsing: Clustering analysis helps to categorize web documents for providing better results for the search target [6].
- Cancer Research: Clustering helps to partition the patients into subgroups with the similar gene expressions. These subgroups can help with better understanding of the disease as well as diagnostic purposes [1].
- City Planning: Clustering can be used to group houses according to their value and location [1].

Clustering methods try to discover the hidden features in the data, group data points based on the similarities of the identified features while optimizing an objective function [7,8,9]. Several clustering methods have been developed [3,9] such as K-means, Fuzzy c-means, mixture models, and spectral clustering. The focus of our study is on the class of K-means clustering methods including K-means and kernel K-means. K-means is a well known clustering method that is commonly used for clustering analysis. It is based on minimizing Euclidean distance between each point and the center of the cluster to which it belongs. The advantages of K-means are its simplicity and speed [10,11,12,13,14].

K-means can discover clusters that are linearly separable. Kernel K-means is a non-linear extension of K-means clustering method. Kernel K-means clustering, as the name implies, uses a kernel function to project nonlinearly separable clusters into a higher-dimensional feature space to make them linearly separable. In our previous work [15], we introduced the idea of combining clustering results of different kernels given the true labels of the entire dataset, i.e., assuming a fully supervised method. This assumption, of course was essentially hypothetical and was only considered to demonstrate proof of concept for the proposed idea.

Because the assumption of knowing the labels for the entire dataset is not true for any real world machine learning application, we extended the work to be applicable to real problems. The proposed clustering method here, aggregates the clustering results obtained using several kernels. To aggregate the clustering results, we develop a weighting function based on normalized mutual information (NMI) score [16,17,18,19] where NMI is computed for clustering results (of a training set with known labels) obtained by different kernels.

### 1.1. Clustering Challenges

There are several challenges associated with data clustering. A major issue is clustering data points that are not linearly separable. A common approach is to project the data points to a different feature space. This is a sound solution. However, the challenge is choosing the relevant transform function. In real world applications, the true groups are unknown. Hence, there is not an obvious choice among transforming functions. To address this issue, one may apply a set of different transform functions, cluster the transformed data points, and aggregate the clustering results. The next related challenge is aggregating the results. Our potential solution to this issue is combining the results using a weight function. The question is: How do we weigh the results? To address this challenge, we propose to use a training set with known labels. First, we cluster the training set. We then evaluate the clustering performance of the training set using the known true labels. The weights are assigned based on the evaluated clustering performance of each transform function. We should point out that kernel K-means as a clustering algorithm is an unsupervised learning method and hence, the proposed aggregated kernel clustering algorithm is essentially an unsupervised method. However, our performance weighting function is based on NMI which is a supervised learning method for evaluation of the clustering performance. It means we use a supervised performance measure along with an unsupervised learning method. Our future work is focused on replacing the supervised performance measure with an unsupervised one and make the whole algorithm unsupervised.

### 1.2. Related Data

To evaluate the performance of the proposed method, four datasets are used. We apply kernel K-means using three different kernels to group he following datasets. Each dataset is then corrupted with low, moderate, and high noise levels and kernel K-means is applied to the noisy data. The datasets are summarized below.

- Two Inner Circles;
  - Noiseless;
  - Corrupted with low noise;
  - Corrupted with moderate noise;
  - Corrupted with high noise;
- Two Moons (half rings);
  - Noiseless;
  - Corrupted with low noise;
  - Corrupted with moderate noise;
  - Corrupted with high noise;
- Iris data;
- DNA copy number data.

## 2. Methods

An overview of k-means and kernel K-means is presented first. Next, we discuss the proposed weighting method using normalized mutual information (NMI) which is calculated to evaluate clustering performance [4,16,17,18,19].

### 2.1. Brief Review of K-Means Clustering

#### 2.1.1. K-means

K-means identifies K centers by minimizing the total squared distance between each point and its assigned cluster center. K-means technique can be summarized by first selecting K arbitrary centers, which are usually, as Lloyd’s algorithm suggests, uniformly selected at random from the data. Second, each point is assigned to a cluster that is the closest to it based on the Euclidean distance between them. Third, a new cluster center is calculated based on the average distance of all data points that belong to that cluster. Finally, the second and third steps are repeated until the algorithm converges. K-means objective function can be written as $\sum_{j=1}^{k}\sum_{x_{a}\in C_{j}}{∥x_{a}-m_{j}∥}^{2}$ , where $C_{j}$ is the cluster *j*, $m_{j}$ is the center of cluster *j*, and $∥.∥$ is the Euclidean distance.

#### 2.1.2. Kernel K-Means

Kernel K-means was introduced to identify clusters that are not linearly separable. The idea of kernel K-means clustering relies on transforming the data into a higher-dimensional feature space using a nonlinear function to make the data points linearly separable in the projected space. Let $\left\{x_{1},x_{2},\cdots ,x_{n}\right\}$ be a set of data points, *k* be the number of clusters, $C_{j}$ be the cluster *j*, ${\left\{C_{j}\right\}}_{j=1}^{k}$ be a partitioning of data points, and ϕ be a non-linear function. Kernel K-means algorithm is summarized below [4].

1. Form the kernel matrix K by calculating its elements. Each element of K is a dot-product in the kernel feature space:
  (1) $$\kappa \left(x_{a},x_{b}\right)=\phi \left(x_{a}\right)\cdot \phi \left(x_{b}\right),\;a,b=1,2,\cdots ,n.$$
  where $\phi (x_{a})$ denotes the data point $x_{a}$ in transformed space. The dot product $\phi \left(x_{a}\right)\cdot \phi \left(x_{b}\right)$ is computed using kernel function κ. Some popular kernel functions are Gaussian, polynomial, and Sigmoid [20].
2. Randomly initialize each cluster center.
3. Compute Euclidean distance of each data point from the cluster center $m_{j}$ in the transformed space:
  (2) $$\phi \left(x_{a}\right)-m_{j}=\phi \left(x_{a}\right)-\sum_{x_{b}\in C_{j}}\frac{\phi (x_{b})}{|C_{j}|}=\phi \left(x_{a}\right)\cdot \phi \left(x_{a}\right)-\frac{2\sum_{x_{b}\in C_{j}}\phi \left(x_{a}\right)\cdot \phi \left(x_{b}\right)}{|C_{j}|}+\frac{2\sum_{x_{b},x_{c}\in C_{j}}\phi \left(x_{b}\right)\cdot \phi \left(x_{c}\right)}{|C_{j}{|}^{2}}$$
  where $|C_{j}|$ is the number of elements in the cluster $C_{j}$.
4. Assign data points to a cluster with minimum distance.
5. Compute the new cluster centers $m_{j},j\in 1,2,\cdots ,k$ as the average of the points belong to cluster $C_{j}$ in transformed space:
  (3) $$m_{j}=\sum_{x_{b}\in C_{j}}\frac{\phi (x_{b})}{|C_{j}|},\;j=1,2,\cdots ,k$$
6. Repeat from step 3 until the objective function is minimized:
  (4) $$\underset{m_{j}}{\mathrm{argmin}}D\left({\left\{C_{j}\right\}}_{j=1}^{k}\right)=\underset{m_{j}}{\mathrm{argmin}}\sum_{j=1}^{k}\sum_{x_{a}\in C_{j}}{∥\phi \left(x_{a}\right)-m_{j}∥}^{2}$$

### 2.2. Aggregated Kernel Clustering

In kernel K-means method, the clustering result of a particular dataset depends on the selected kernel function. Several kernel functions have been introduced for kernel clustering such as Gaussian, polynomial, linear, spline, and hyperbolic tangent. Selecting a kernel function for a clustering application is a challenging task. Hence, one may use a set of different kernel functions, perform kernel K-means using each kernel, and combine the clustering results.

A common approach to combine the clustering results is majority voting where the cluster label of each object is decided by the majority and therefore often an odd number of clustering results are combined by this method. For example, assume three clustering methods group object $x_{a}$ in cluster 1 and two clustering methods group $x_{a}$ in cluster 2. Because there are 3 votes for cluster 1 and 2 votes for cluster 2, the majority voting method will cluster $x_{a}$ to group 1. In this way, each method gets the same weight regardless of their performance.

To address this issue, we aggregate the results of several kernel functions by proposing Weighted Mutual Information (WMI). The idea of WMI is appealing, because it will address the shortcoming of majority voting by introducing the concept of "weights" based on clustering performance. Therefore, to combine the results, we first calculate mutual information to quantify the performance of each method. Computing the performance weights (WMIs) from partially labeled data is based on supervised learning concept assuming that a training set with true class labels is available.

#### 2.2.1. Normalized Mutual Information

Mutual Information (MI) is defined by:

(5) MI=H(T)−H(T|C)

where H(T) is entropy of true class labels *T*:

(6) $$H\left(T\right)=-\sum_{i=1}^{t}P\left(T_{i}\right)log\left(P\left(T_{i}\right)\right)$$

where *t* is the number of true classes and $T_{i}$ is true class *i*. H(T|C) is conditional entropy of true class labels *T* given clustering result *C*, i.e., the entropy of class labels within each cluster:

(7) $$H\left(T|C_{j}\right)=-P\left(C_{j}\right)\sum_{i=1}^{t}P\left(T_{i}|C_{j}\right)log\left(P\left(T_{i}|C_{j}\right)\right)$$

where $C_{j}$ is cluster *j* in the clustering result. Although, MI can be used to evaluate the clustering result, it is not bounded. To compare the clustering results obtained by different methods, we prefer to use a criterion with specific bounds. To put bounds on MI, it can be normalized. Normalized Mutual Information (NMI) is obtained by normalizing MI using entropy of the true class labels and the clustering result as follow:

(8) $$\eta =\frac{2MI}{H(T)+H(C)}=\frac{2(H(T)-H(T|C))}{H(T)+H(C)}$$

where η is NMI, and H(C) is:

(9) $$H\left(C\right)=-\sum_{j=1}^{c}P\left(C_{j}\right)log\left(P\left(C_{j}\right)\right)$$

where *c* is the number of clusters in the clustering result. For a perfect clustering result where t=c, $T_{i}=C_{j}$, and so H(C)=H(T). Moreover, $P(T_{i}|C_{j})=1$, and hence $log(P\left(T_{i}|C_{j}\right))=0$, H(T|C)=0, and in turn:

(10) $$\eta =\frac{2MI}{H(T)+H(C)}=\frac{2H(T)}{2H(T)}=1$$

**Figure 1:**
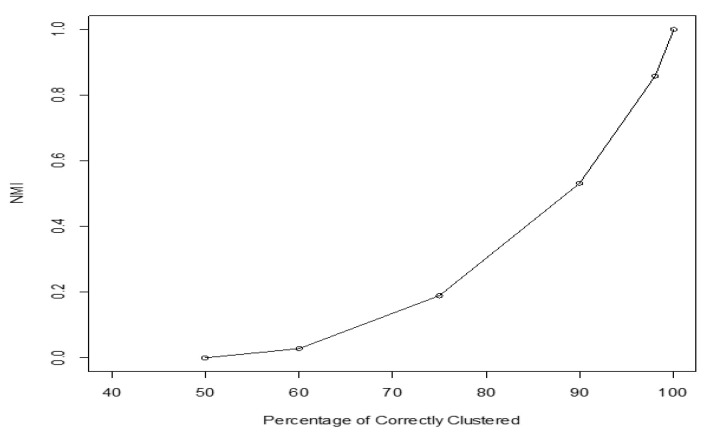
NMI (Normalized Mutual Information) score vs. true positive rate (percentage of elements clustered in right groups).

NMI values close to one indicate that most of identified cluster labels agree with the true class labels. That is, most of the objects that belong to the same class are clustered in the same cluster [4]. NMI value ranges from zero to one, but we should point out that it is a non-linear criterion for the clustering performance. For example, if in the clustering result, half of the data is correctly clustered, a linear criterion will score 0.5, while NMI score is zero. Figure 1 shows NMI values with regard to clustering performance. It shows that NMI has a value of zero when 50% of the elements are correctly clustered, a value of about 0.5 when 88% of the elements are correctly clustered, a value of 0.6 when 93% of the elements are correctly clustered, and a value of one when 100% of the elements are correctly clustered.

#### 2.2.2. Weighted Mutual Information (WMI)

Assume *R* different kernel functions are used to generate *R* clustering results. Let $\eta_{r}$ be obtained NMI score using a training set for clustering result r∈1,2,⋯,R:

(11) $$\eta_{r}=\frac{2MI_{r}}{H(T)+H(Cr)}=\frac{2(H(T)-H(T|Cr))}{H(T)+H(Cr)}$$

where H(Cr) is the entropy of clustering result *r*, $MI_{r}$ is MI regarding clustering result *r*, and $\eta_{r}$ is NMI of clustering result *r*. We define Weighted Mutual Information (WMI) or performance weight of clustering result *r* by:

(12) $$w_{r}=\frac{\eta_{r}}{\sum_{k=1}^{R}\eta_{k}}=\frac{\frac{2MI_{r}}{H(T)+H(Cr)}}{\sum_{k=1}^{R}\frac{2MI_{k}}{H(T)+H(Ck)}}=\frac{\frac{H(T)-H(T|Cr)}{H(T)+H(Cr)}}{\sum_{k=1}^{R}\frac{H(T)-H(T|Ck)}{H(T)+H(Ck)}},\;r=1,2,\cdots ,R.$$

and $\sum_{r=1}^{R}w_{r}=1$. H(T), H(C), and H(T|C) are computed by:

(13) $$H\left(T\right)=-\sum_{i=1}^{t}\frac{n_{i}}{n}log\left(\frac{n_{i}}{n}\right),$$

(14) $$H\left(C\right)=-\sum_{j=1}^{c}\frac{n_{j}}{n}log\left(\frac{n_{j}}{n}\right),$$

and

(15) $$H\left(T|C\right)=\sum_{j=1}^{C}\frac{n_{j}}{n}\sum_{i=1}^{t}\frac{n_{j}^{i}}{n_{i}}log\left(\frac{n_{j}^{i}}{n_{i}}\right)$$

where $n_{i}$ is the number of objects in the true class *i*, $n_{j}$ is the number of objects grouped in the cluster *j*, $n_{j}^{i}$ is the number of objects grouped in the cluster *j* and belong to the true class *i*, and *n* is the total number of objects.

After calculating the performance weights (WMIs) for *R* clustering result, we combine the results obtained by different kernels (different clustering methods) as follow. For a given object $x_{a}$, we obtain the assigned group label by each kernel and compute:

(16) $$w_{j}^{x_{a}}=\sum_{r=1}^{R}w_{r}|\left(x_{a}\in C_{j}\right)$$

where $w_{r}$ is the performance weight (WMI) of the method *r* and

(17) $$w_{r}|\left(x_{a}\in C_{j}\right)=w_{r}\;\mathrm{if}\;x_{a}\in C_{j},$$

and it is equal to zero if $x_{a}\notin C_{j}$. The cluster label for object $x_{a}$ will then be determined by:

(18) $$\underset{j}{\mathrm{argmax}}w_{j}^{x_{a}}$$

## 3. Results

Two datasets including two inner circles and two moons are two-dimensional, Iris data is four-dimensional, and DNA copy number data is several thousand-dimensional. In the first step, kernel K-means method is applied to a training set with known labels. Three different kernels including Gaussian, polynomial, and hyperbolic tangent are used. NMI score is computed for clustering result obtained by each kernel for the training set. For each noise level and each kernel, clustering results for 100 different instances of noisy data are obtained and NMI scores are computed. Monte Carlo average NMI is then computed. The Monte Carlo average NMI score obtained by kernel *r* (for the training set corrupted with specific noise level) will be used to calculate the weight of the kernel *r*. Next, we use the kernels to group the whole dataset. The clustering results are then aggregated by the weights that were calculated for the training set in the previous step. Finally, we compare the aggregated results obtained by majority voting and WMI.

### 3.1. Two Inner Circles

**Figure 2:**
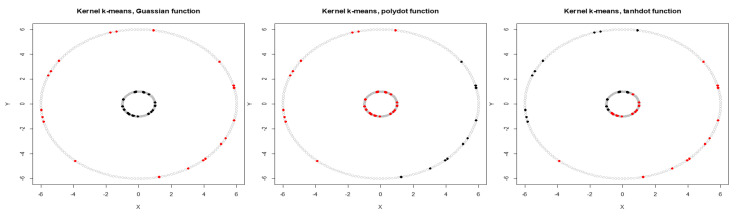
Clustering of the labeled training set for two inner circles (gray dots show entire data) obtained by Gaussian, polynomial, and tangent kernels.

**Figure 3:**
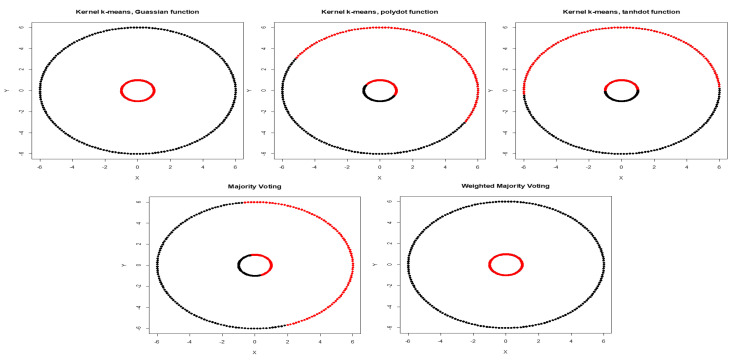
Clustering results obtained by Gaussian, polynomial, and tangent kernels along with aggregated results obtained by majority voting and WMI (Weighted Mutual Information) kernel clustering.

We first calculate the average NMI score and estimate WMI for each kernel using partially labeled data. The clustering results and estimated WMIs are summarized in Table 1. For the noiseless inner circles, among all kernels, Gaussian kernel has the best performance evaluated by average NMI score. The clustering results are then combined using WMI obtained for each kernel. Gaussian kernel gets the highest weight of 0.740. Figure 2 shows the clustering results of training data marked in black and red obtained by three different kernels. Training data is a subset with known labels that is randomly selected from the original true groups (marked in light gray). Among the three kernels, Gaussian is able to completely separate the two original clusters. Table 2 summarizes the clustering results obtained using Gaussian, polynomial, and tangent kernels along with combined results using majority voting and the proposed WMI. The first row of the table shows NMI score for Gaussian (score of one), polynomial (score of zero), and hyperbolic tangent kernel (score of zero). The results indicate that polynomial and hyperbolic tangent kernels with NMI of zero can cluster only half of the data points into the right clusters. WMI scores (Table 1) are calculated for the training data and are used for aggregating the results. As we can see in Table 2, the WMI method performs better than majority voting. The visual representation of clustering result (Figure 3) obtained by WMI and majority voting show how WMI is able to detect the original classes while majority voting suffers by giving equal votes to each kernel.

**Figure 4:**
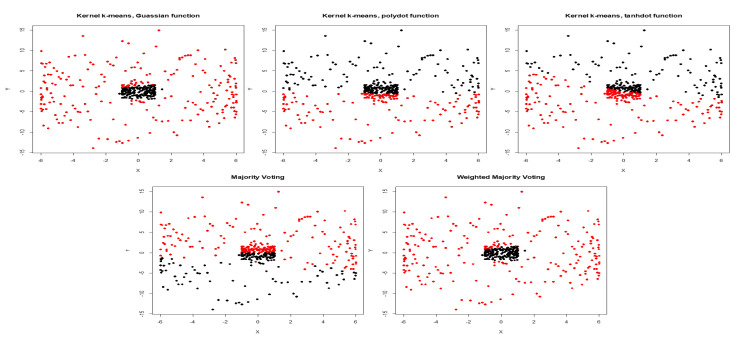
Clustering results obtained by Gaussian, polynomial, and tangent kernels along with aggregated results obtained by majority voting and WMI kernel clustering for inner circles corrupted with high noise.

Next, we added low level of noise to the inner circles. With low noise, Gaussian kernel has the highest weight of 0.722, while the weights for polynomial and tangent kernels are 0.248 and 0.029 respectively (Table 1). Figure 4 shows clustering results obtained by three different kernels, and aggregated result obtained using majority voting and WMI for inner circles corrupted with high noise. Aggregated result using WMI is impartial and yields an NMI score of 0.74. It means the weighted method not only outperforms majority voting, but also performs better than Gaussian kernel that had the best performance among all kernels with NMI score of 0.71 (Table 2).

### 3.2. Two Moons

**Figure 5:**
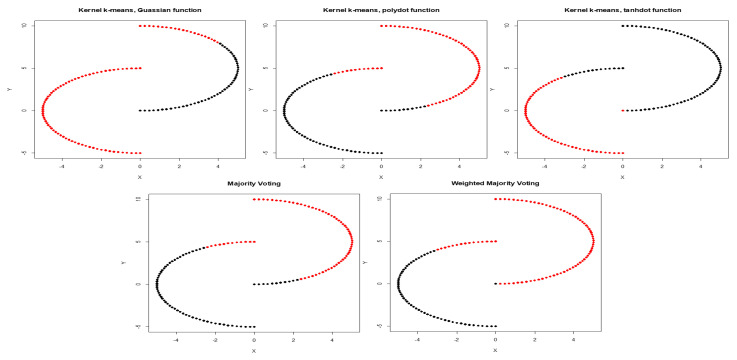
Clustering results obtained by Gaussian, polynomial, and tangent kernels along with aggregated results obtained by majority voting and WMI kernel clustering for Two Moons.

**Figure 6:**
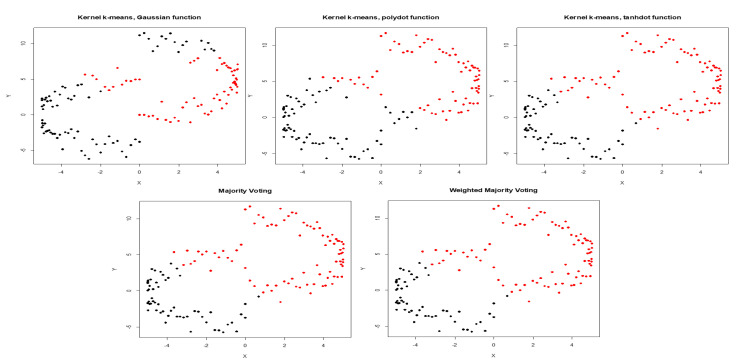
Clustering results obtained by Gaussian, polynomial, and tangent kernels along with aggregated results obtained by majority voting and WMI kernel clustering for Two Moons corrupted with high noise.

The average NMI score along with WMI value of each kernel are computed for a training set (with known labels) and are summarized in Table 3. The clustering results for noiseless two moons data and two moons data corrupted with high noise are depicted in Figure 5 and Figure 6 respectively. Based on NMI scores, tangent kernel performs better than the other kernels for clustering two moons data. The weight of the tangent kernel obtained for the training set is the highest among all kernels. The calculated NMI of the proposed method (WMI) for the clustering of two moons corrupted with low noise is 0.55 in comparison with NMI score of 0.48 yields by majority voting (Table 4). Because NMI is a non-linear criterion, 0.55−0.48=0.07 is a substantial difference between the NMI scores (Figure 1).

### 3.3. Iris

**Figure 7:**
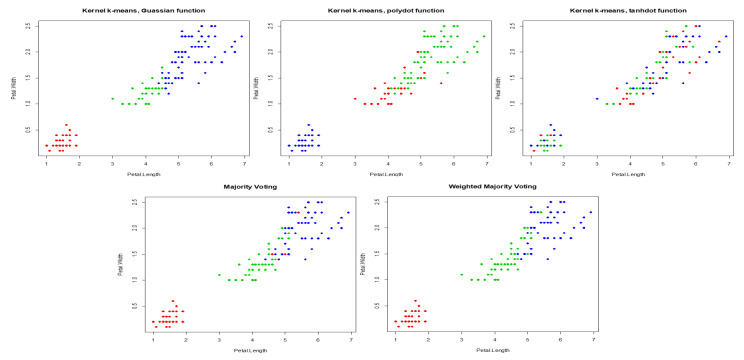
Clustering results obtained by Gaussian, polynomial, and tangent kernels along with aggregated results obtained by majority voting and WMI kernel clustering for Iris data.

We applied the proposed method to Iris data, a 4-dimensional dataset with three classes. A training set is randomly selected to calculate NMI score and weight of each kernel. For Iris data, polynomial kernel performs better than the Gaussian and tangent kernels (Table 5). WMIs are 0.429, 0.505, and 0.066 for Gaussian, polynomial, and tangent respectively. The aggregated result obtained by WMI (Figure 7) yields an NMI score of 0.725 which is higher than the NMI score of 0.58 obtained by majority voting (Table 6).

### 3.4. Application to DNA Copy Number Data

**Figure 8:**
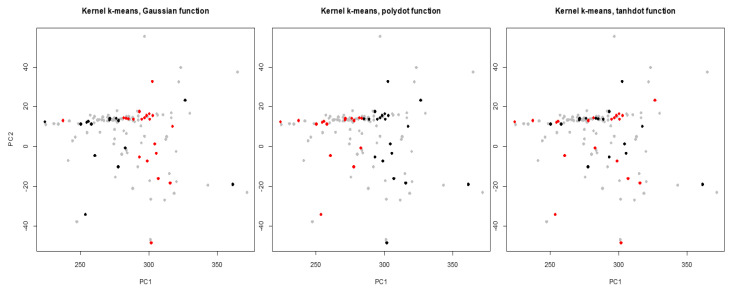
Clustering of the labeled training set for chromosome one (gray dots show entire data) obtained by Gaussian, polynomial, and tangent kernels.

**Figure 9:**
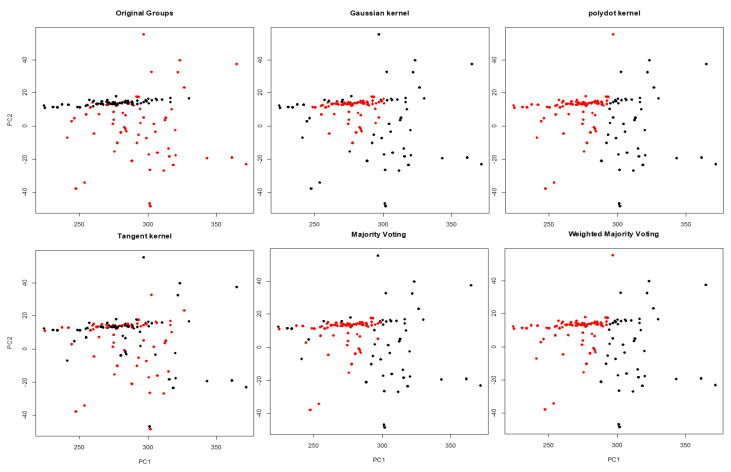
Clustering results obtained by Gaussian, polynomial, and tangent kernels along with aggregated results obtained by majority voting and WMI kernel clustering for chromosome one.

Lung cancer is the leading cause of cancer death among both men and women, making up almost 25% of all cancer deaths and early diagnosis increases the chance of patient survival. Hence, it is important to recognize lung cancer from the non-involved tissue in the early stage of cancer. A potential approach is to use similarities and disparities between cancer and control (paired peripheral blood) samples [21,22]. The dataset contains DNA copy numbers obtained for paired cancer-control samples of 63 early stage non-small cell lung cancer patients. We applied the proposed WMI kernel clustering method to group the DNA copy numbers of chromosome one into two groups. Let $\left\{x_{1},x_{2},\cdots ,x_{126}\right\}$, $x\in R^{m}$, be a set of 126 subjects including cancer and matched blood samples for each patient (total of 63 patients), where *m* is the number of features for the chromosome. We should point out that, this is a very challenging clustering task because the data is several-thousand dimensional. There are 19,873 obtained copy numbers (features) for cancer and blood samples in the chromosome one of each patient. For the visualization of the clustering results, the first and the second principal components are selected. First, NMI score and WMI value are computed for clustering of a training set by each kernel. The clustering results are depicted in Figure 8, and NMI scores and WMI values are summarized in Table 7. As we can see in Table 7, polynomial kernel performs better than the other kernels based on NMI score. The obtained weight by the polynomial kernel for the training set is 0.762 which is the highest among all kernels. Aggregated result using WMI yields an NMI score of 0.075 and outperforms majority voting (Table 8). Figure 9 shows that the clustering results obtained by the proposed method (bottom right corner) is closer to the true classes (top left corner) than the majority voting.

## 4. Conclusions

An important task in machine learning is dividing data into different groups. K-means and its extensions are broadly used for cluster analysis. While K-means can identify the groups that are linearly separable, kernel K-means has been introduced to separate the clusters that are not linearly separable. Kernel K-means projects the data points to a new feature space using a transforming function. Different kernel functions do not perform at the same level when they are applied to cluster a dataset. Therefore, choosing the right kernel for an arbitrary dataset is a challenging task. To address this issue, one can apply a set of different kernels and aggregate the results. In this study, we introduced Weighted Mutual Information (WMI) to combine the clustering results obtained by different transforming functions. The performance weights (WMIs) are calculated based on performance of each transform function for clustering of a training set. We first calculate WMI for each kernel using its NMI score. Then, we cluster the entire dataset using the same set of kernel functions. Next, we aggregate the clustering results using the calculated WMI for each kernel. The proposed method provides an impartial performance regardless of choice of transforming function. The combined result is rather obtained by collective performance of all kernel functions. For example, in clustering of Iris data, the performance of polynomial function is better than Gaussian kernel and as such it has a higher WMI. However, clustering performance of polynomial function is lower than that of Gaussian for clustering of the entire Iris data. Regardless of the inconsistent performance of polynomial function to cluster this dataset, the aggregated kernel result is comparable with the highest WMI yielded by Gaussian kernel for clustering of the entire Iris dataset. In contrast in clustering of copy number data, the performance of polynomial function is better than Gaussian and tangent kernels for both training set and entire data. However, tangent kernel performs better than Gaussian for clustering of the training set, but it performs worse than Gaussian for clustering of the entire copy number data. Again, regardless of the inconsistent performance of different kernels to cluster copy number data, the aggregated kernel result is impartial and comparable with the highest WMI yielded by polynomial kernel for clustering of the entire dataset. Overall, the proposed WMI can potentially improve the clustering result specially in high noise.

### How to Handle Undersampled Data, How to Select k, and How to Select a Kernel?

To choose the number of clusters k, kernel K-means will be performed for different values of k by varying k from 1 to K for each kernel separately. Total within-cluster sum of square (WSS) will be calculated for each kernel and each value of k. The average WSS (AWSS) of all kernels for each value of k will then be obtained. Plot the AWSS curve with regard to the number of clusters k. The value of k that provides the minimum AWSS (MAWSS) will be chosen. For undersampled data, in place of Equations (13) to (15), NSB (Nemenman, Shafee, and Bialek) algorithm [23] can be used for estimation of entropy. We should point out that, the main motivation to implement the proposed method is that choosing the relevant kernel for an arbitrary application is challenging. Therefore, in place of relying and justifying the use of a specific kernel for the application at hand, we propose to use a pool of kernels and aggregate the results obtained by different kernels in the pool. 

## Figures and Tables

**Table 1:** NMI (Normalized Mutual Information) scores and WMI (Weighted Mutual Information) values obtained by different kernels for clustering of a training set (with known labels) randomly selected from inner circles data corrupted with different levels of noise.

		Gaussian Kernel	Polynomial Kernel	Tangent Kernel
Noiseless	NMI	1	0.344	0.008
	WMI	0.740	0.254	0.006
Low Noise	NMI	0.698	0.240	0.028
	WMI	0.722	0.248	0.029
Moderate Noise	NMI	0.678	0.238	0.027
	WMI	0.719	0.252	0.029
High Noise	NMI	0.602	0.216	0.03
	WMI	0.710	0.254	0.036

**Table 2:** Performance of clustering results (evaluated by NMI score) obtained by different kernels along with majority voting and WMI kernel clustering for inner circles corrupted with different noise levels.

	Gaussian Kernel	Polynomial Kernel	Tangent Kernel	Majority Voting	WMI Kernel Clustering
Noiseless	1	0	0	0.001	1
Low Noise	0.799	0.020	0.0003	0.176	0.801
Moderate Noise	0.821	0.140	0.001	0.162	0.810
High Noise	0.713	0.154	0.002	0.175	0.742

**Table 3:** NMI scores and WMI values obtained by different kernels for clustering of a training set (with known labels) randomly selected from two moons data corrupted with different levels of noise.

		Gaussian Kernel	Polynomial Kernel	Tangent Kernel
Noiseless	NMI	0.353	0.241	0.769
	WMI	0.259	0.177	0.564
Low Noise	NMI	0.285	0.238	0.576
	WMI	0.259	0.216	0.524
Moderate Noise	NMI	0.31	0.232	0.568
	WMI	0.279	0.209	0.512
High Noise	NMI	0.322	0.208	0.531
	WMI	0.303	0.196	0.501

**Table 4:** Performance of clustering results (evaluated by NMI score) obtained by different kernels along with majority voting and WMI kernel clustering for Two Moons corrupted with different noise levels.

	Gaussian Kernel	Polynomial Kernel	Tangent Kernel	Majority Voting	WMI Kernel Clustering
Noiseless	0.333	0.372	0.551	0.482	0.55
Low Noise	0.337	0.230	0.559	0.464	0.551
Moderate Noise	0.322	0.224	0.532	0.473	0.526
High Noise	0.336	0.373	0.479	0.436	0.479

**Table 5:** NMI scores and WMI values obtained by different kernels for clustering of a training set (with known labels) randomly selected from Iris data.

		Gaussian Kernel	Polynomial Kernel	Tangent Kernel
Iris Data	NMI	0.765	0.899	0.117
	WMI	0.429	0.505	0.066

**Table 6:** Performance of clustering results (evaluated by NMI score) obtained by different kernels along with majority voting and WMI kernel clustering for Iris data.

	Gaussian Kernel	Polynomial Kernel	Tangent Kernel	Majority Voting	WMI Kernel Clustering
Iris Data	0.732	0.696	0.006	0.582	0.725

**Table 7:** NMI scores and WMI values obtained by different kernels for clustering of a training set (with known labels) randomly selected from DNA copy number data for chromosome one.

		Gaussian Kernel	Polynomial Kernel	Tangent Kernel
Chromosome Data	NMI	0.002	0.037	0.009
	WMI	0.048	0.762	0.189

**Table 8:** Performance of clustering results (evaluated by NMI score) obtained by different kernels along with majority voting and WMI kernel clustering for chromosome one in DNA copy number dataset.

	Gaussian Kernel	Polynomial Kernel	Tangent Kernel	Majority Voting	WMI Kernel Clustering
Chromosome Data	0.054	0.075	0.012	0.064	0.075
